# Financial Toxicity in Head and Neck Cancer Patients Treated With Proton Therapy

**DOI:** 10.14338/IJPT-20-00054.1

**Published:** 2021-06-25

**Authors:** Grace L. Smith, Ya-Chen Tina Shih, Steven J. Frank

**Affiliations:** 1 Department of Radiation Oncology, The University of Texas MD Anderson Cancer Center, Houston, TX, USA; 2 Department of Health Services Research, The University of Texas MD Anderson Cancer Center, Houston, TX, USA

**Keywords:** financial toxicity, head and neck cancer, proton

## Abstract

Cancer-related financial toxicity impacts head and neck cancer patients and survivors. With increasing use of proton therapy as a curative treatment for head and neck cancer, the multifaceted financial and economic implications of proton therapy—dimensions of “financial toxicity”—need to be addressed. Herein, we identify knowledge gaps and potential solutions related to the problem of financial toxicity. To date, while cost-effectiveness analysis has been used to assess the value of proton therapy for head and neck cancer, it may not fully incorporate empiric comparisons of patients' and survivors' lost productivity and disability after treatment. A cost-of-illness framework for evaluation could address this gap, thereby more comprehensively identifying the value of proton therapy and distinctly incorporating a measurable aspect of financial toxicity in evaluation. Overall, financial toxicity burdens remain understudied in head and neck cancer patients from a patient-centered perspective. Systematic, validated, and accurate measurement of financial toxicity in patients receiving proton therapy is needed, especially relative to conventional photon-based strategies. This will enrich the evidence base for optimal selection and rationale for payer coverage of available treatment options for head and neck cancer patients. In the setting of cancer care delivery, a combination of conducting proactive screening for financial toxicity in patients selected for proton therapy, initiating early financial navigation in vulnerable patients, engaging stakeholders, improving oncology provider team cost communication, expanding policies to promote price transparency, and expanding insurance coverage for proton therapy are critical practices to mitigate financial toxicity in head and neck cancer patients.

## Introduction

Though technological and biomedical advances in cancer therapies have propelled the improvement of key oncology patient outcomes—survival, cancer progression, and treatment-related toxicity—cancer treatment, often costly, is a source of financial hardship for many cancer patients and their families [1, 2]. Cancer-related financial hardship, or “financial toxicity,” impacts as many as half of cancer patients. Financial toxicity encompasses the burdens on patients resulting from medical costs of cancer treatment, side-effect management, and survivorship care, along with such nonmedical costs as the extra transport and housing costs often needed to access cancer treatment [1–3]. Additional financially impactful consequences of cancer are patient job loss, decreased work productivity, disability, inability to care for dependents, accumulated medical debt, and bankruptcy [2, 4–13]. The “toxicity” of cancer-related financial burdens is reflected in subsequent adverse health outcomes, with worse quality of life, decreased psychosocial well-being, and lower treatment adherence reported by patients experiencing cancer-related financial hardship [2, 10, 11].

Though cancer-related financial toxicity impacts patients across the spectrum of diseases, evidence suggests that there are particular aspects of financial toxicity that affect head and neck cancer patients, who make up an estimated 65 000 new cases annually in the United States and 550 000 annual cases worldwide. Only a small number of studies have focused solely on the financial hardship in head and cancer patients, but the limited evidence suggests that approximately one third of head and neck cancer patients report between moderate and catastrophic levels of financial hardship [14, 15]. A deeper understanding of the sources of financial hardship in head and neck cancer patients is needed to design interventions and develop strategies to mitigate this problem. Studies of cancer patients across the spectrum of disease sites reveal several common risk factors for financial hardship: having a lack of health insurance or being underinsured, having lower income or a change in employment, living alone, and being younger (working) age [2, 15]. In addition, several factors especially relevant to the treatment of head and neck cancer patients could further aggravate financial hardship. Longer duration and greater intensity of cancer treatment, both aspects of multimodality treatment, are associated with higher risks of patients experiencing financial hardship [2]. Given that radiation treatment is a foundational component of curative multimodality therapy for head and neck cancers, the consideration of financial toxicity in head and neck patients receiving radiation treatment has garnered increasing attention [15–17].

## Addressing the Economic Impact of Proton Therapy

With the rapidly evolving and increasingly implemented strategy of proton therapy for head and neck cancer, concurrent questions have arisen on how to address the economic impact of proton therapy. This economic impact is, undeniably, not only an individual patient concern but also a societal concern. With the costs of cancer care rising—annually, an estimated financial burden of $80 billion on the US healthcare system—calls abound to achieve higher value in cancer care, by developing and selecting treatments that optimize clinical outcomes yet minimize economic burdens, the critical component of value in care [18–20]. Yet one fundamental conundrum for biomedical advances in oncology, also specifically relevant to advanced technology in radiation treatments such as proton therapy, is that while novel treatments hold promise for improving outcomes in cancer patients, the clinical promise is often accompanied by substantially higher costs. Resolving this conundrum requires assessing marginal gains in value by comprehensively identifying how different treatment options impact all components of value to patients and to society. This information is additive to the knowledge of the clinical comparative effectiveness of treatment options. This comprehensive value assessment in care is the present, pressing challenge for advancing new technologies and other novel therapies in oncology practice. If successful, identifying treatments that enhance value in acute and long-term follow-up will help preserve the financial resilience of health care systems—and of individual cancer patients, survivors, and their families.

A primary aspect of the clinical comparative effectiveness of proton therapy is the capability of the physical properties of the Bragg peak to decrease normal tissue dose and, in turn, decrease normal tissue toxicity risks compared with conventional photon therapy. The need for continuing improvement of the toxicity profile of radiation treatment for head and neck cancer is evident. Prior studies, mainly dominated by patients treated with conventional photon treatment, demonstrate the high frequency of acute side effects, including as many as 92% of patients who experience radiation-associated mucositis and 26% with severe dehydration. There are also long-term, debilitating side effects— for example, 29% with xerostomia, 26% to 57% with dysphagia, 20% with dysgeusia, and 9% with a feeding tube [21–25]. These toxicities represent a major source of morbidity in head and neck cancer patients impacting quality of life and function.

Moreover, these toxicities and chronic survivorship issues contribute to excess health care costs in head and neck cancer patients. In a recent study, Massa and colleagues [26] used a national data source, the Medical Expenditure Panel Survey, to analyze 16 771 US cancer patients. Compared with patients with other cancers, head and neck cancer patients not only were more like to be a racial/ethnic minority, poorer, or less educated and to have lower health status but were also more likely to incur higher total health care costs and out-of-pocket medical costs. The median annual medical expenses for patients with head and neck cancer was approximately $8000 in this study, compared with approximately $6000 in patients with other cancers. The costliness of care on the health care system was also passed on to individual patients, as head and neck cancer patients and survivors in this cohort spent nearly 4% of their annual income on medical expenses [26].

Besides the direct medical expenses related to illness, functional disabilities in head and neck cancer patients appear to be a risk factor that may contribute to a high frequency of missed and lost work with cancer treatment; an estimated half of head and neck cancer patients face persistent barriers to resuming employment [27, 28]. The loss of employment and work productivity after a diagnosis of head and neck cancer is not only a threat to individual patients, which could contribute to financial toxicity, but also a major societal loss. Such economic and financial toxicity implications therefore underscore the importance of identifying therapeutic strategies that can mitigate short-term and long-term treatment-related physical toxicity as a strategy to mitigate financial toxicity.

Accordingly, examinations of the clinical benefit and comparative toxicity profile of proton therapy in head and neck cancer treatment are directly relevant to developing approaches to mitigate financial toxicity. One such examination was a case-matched study of 50 oropharyngeal cancer (OPC) patients treated definitively with intensity modulated proton therapy (IMPT) and 100 patients with intensity modulated photon therapy (IMRT) combined with concurrent chemotherapy. Blanchard et al [29] demonstrated a reduction of the composite endpoint of grade 3 weight loss or gastrostomy (G)-tube presence associated with IMPT, with the likelihood of these adverse outcomes reduced at the 3-month follow-up (odds ratio [OR] = 0.44; 95% confidence interval [CI]: 0.19–1.0; *P* = .05). The benefit persisted also at the 1-year follow-up (OR = 0.23; 95% CI: 0.07–0.73; *P* = 0.01) [29]. Sio et al [30] reported lower head and neck symptom burden through a 3-month follow-up in OPC patients treated with IMPT. Similarly, a favorable toxicity profile for OPC patients after IMPT was reported by Aljabab et al [31], including those treated in a postoperative setting. A recent seminal analysis was reported on 1483 cancer patients (of all disease sites, including head and neck cancer patients), 391 of whom received proton therapy and 1092 photon therapy with concurrent chemotherapy. In this study, Baumann et al [32] found that patients who received proton therapy had fewer grade 2 and grade 3 adverse events leading to 90-day unplanned acute hospitalizations. Given that acute events such as unplanned hospitalizations and emergency visits represent one of the costliest aspects of cancer care [33], these findings suggest that the potential clinical benefits of proton therapy could potentially translate into benefits for cost, health care delivery, and the health care system.

Nevertheless, the economic value of proton therapy for the treatment of head and neck cancer remains debated. At the heart of this debate is the high cost of delivering proton therapy; it requires as much as double the expenditures for capital investment, quality assurance, and operations along with radiation delivery [34]. Accordingly, a recent model by Sher and colleagues [35] demonstrated challenges to cost-effectiveness of proton therapy compared with IMRT, examined using a Markov model approach, in the use case of locally advanced OPC treated with chemoradiation. In this model, the incremental cost-effectiveness ratio for using proton therapy versus IMRT was more than $250 000 per quality-adjusted life year (QALY), much higher than thresholds for societal willingness-to-pay metrics of $100,000-$150 000/QALY. The incremental cost-effectiveness ratio was found to be less than $100 000/QALY in the specific scenario of treatment for human papillomavirus–positive younger patients (ie, less than 55 years old), assuming a 50% reduction in xerostomia and gastrostomy use associated with proton therapy, which were the major toxicities assumed to impact patients' health utility in the model. One implication of these data raised by the authors was the need for clinical trials and research resources to rigorously evaluate the clinical effectiveness of proton therapy, especially within younger (working age) patients [35].

A second implication relates to the need to advance the conceptual framework and analytical approach to evaluating the value of proton therapy in head and neck cancer patients beyond measures of cost-effectiveness alone, since simply restricting the development and adoption of proton therapies based on cost-effectiveness metrics is unlikely to be an entirely long-term viable or equitable solution in a mixed-payer system such as the US health care system. Many US payers do not explicitly apply cost-effectiveness analysis in reimbursement decisions. Furthermore, as cost-effectiveness analyses alone do not fully incorporate an empiric comparison of critical aspects of patients' financial hardship—the loss of work and productivity—more data are needed to directly evaluate and compare the effect of proton therapy versus IMRT on patients' labor market participation, paid and unpaid work, and household and societal productivity, as well as on restoring patients' function, thereby identifying the optimal therapeutic strategies for recovering illness and disability-related functional and financial burdens. This approach to evaluation would therefore use a “cost-of-illness” framework to quantify patients' economic burdens [36–38]. Such a framework emphasizes identifying treatment approaches that restore patient *function* and consequently the economic value of therapy to the patient and society, thereby indirectly offsetting higher costs of treatment. Although not yet widely used to compare proton therapy versus IMRT, engaging studies through the lens of this framework would represent a patient-centered lens when comparing cost of illness, a lens that has already been applied for evaluating the value of systemic and targeted therapies for cancer of other disease sites [39, 40]. Our research group, conducting the ongoing randomized trial of proton versus photon treatment for oropharyngeal cancer patients (NCT01893307) [41], is evaluating patients' work and productivity outcomes after chemoradiation as a secondary outcome. As evidence from research studies continues to collectively emerge on the cost of illness in head and neck cancer patients, simultaneously identifying intervenable and actionable targets to reduce the long-term cost of illness is critical.

## Access to and Delivery of Proton Therapy

Several unique aspects of accessing proton therapy also impact patient financial toxicity. Since there are only just over 30 proton therapy centers geographically dispersed across the United States, tending to cluster in metropolitan areas and academic medical centers, for the vast majority of cancer patients, the burden of housing and transportation costs to obtain proton treatment is likely to be higher on average, than for the more widely accessible photon therapy facilities (and facilities delivering systemic therapies, as another benchmark comparator). And while a relative plethora of data exist on the out-of-pocket cost burden from cancer drugs [42], limited contemporary data exist to identify the range of out-of-pocket expenses that patients encounter associated with proton therapy. Also, because barriers to insurance coverage for proton therapy remain persistent [43], for the certain group of patients who ultimately opt to pay for proton therapy as out-of -network care or directly out of pocket after insurance denial, the financial toxicity of treatment may become particularly catastrophic.

The lack of consistent insurance coverage for proton therapy—including coverage in the setting of clinical trials needed to establish Level I evidence for comparative effectiveness—as well as delays in care due to the lengthy approval process may increase the risk of financial toxicity at the patient level [44, 45]. Innovative solutions to coverage barriers have been previously discussed by Bekelman et al [46]. These include coverage for patients under study participation policies, or, at the facility level, reference pricing with or without evidence development (where the price of proton therapy is set as equivalent to IMRT), bundling care-based contracts, or shared risk programs (where the facility itself takes on the financial risk of proton therapy if it is not covered after insurance appeal). One issue often overlooked in coverage decisions for novel treatments is the life cycle of the product, as the costs of new treatments tend to decrease over time. Accurately capturing the value of a new technology, such as proton therapy, requires taking a dynamic approach that incorporates pricing variation in the product life cycle into the assessment of long-term costs and cost-effectiveness. Finally, while barriers to coverage for proton therapy have been recognized as an obstacle to establishing the comprehensive clinical evidence needed to optimize patient selection for proton therapy in multiple disease sites, including head and neck cancer, there is less recognition of the need to establish evidence supporting strategies to mitigate financial toxicity in head and neck cancer patients. Given the vulnerability of head and neck cancer patients to financial toxicity, a key component of evaluating innovative solutions to coverage barriers will be measuring financial toxicity outcomes along with evaluating clinical, economic, and care delivery outcomes.

The aspects of financial toxicity relevant to proton therapy suggest the need to implement proactive strategies for early financial toxicity intervention in care delivery for cancer patients initiating a course of proton therapy. Financial hardship screening and measurement, combined with stakeholder engagement, to channel patients toward early financial navigation is a strategy that has demonstrated early success in pilot studies [47–49]. An important component of this strategy involves engaged communication between patients and the oncology team, though notably in multiple prior studies, cancer patients underscore an unmet need for communication around financial hardship concerns [50, 51]. An extraordinarily challenging aspect of cancer care and financial navigation as well as cost communication in the setting of cancer care delivery is the difficulty of forecasting the cost trajectory of care, and this challenge remains for delivery of proton therapy as well. At a policy level, efforts to promote price transparency and expand coverage for proton therapy (especially in randomized trials) within a third-party payer-based health care system remain of utmost priority for high-level system-based mitigation of financial toxicity [52]. Yu et al [45] advocate for a collaborative approach with payers to establish the appropriate evidence of the comparative effectiveness of proton therapy, which supports not only prioritization of patient selection to maximize treatment benefit—best clinical practice—but also refinement of efficiencies in delivery—best value for treatment. Sharing and advancing the goals of improving use, reducing waste, and optimizing efficiencies in planning and treatment delivery at the payer-facility level, coupled with minimizing financial toxicity at the patient level, are foundational to increasing value of proton therapy for head and neck cancer. These shared goals are influential from societal, patient, payer, and provider perspectives. Given the impact of the extraordinary socioeconomic upheaval and disparities prompted by the recent COVID-19 pandemic, these stressors highlight the immediate and pressing need for implementing interventions to mitigate cancer-related financial toxicity in vulnerable patients [53].

## Conclusion

Patients with head and neck cancer are vulnerable to cancer-related financial toxicity due to high-risk associated sociodemographic factors, along with an intensive, often multimodality therapy strategy needed for cure and the related long-term toxicities of treatment. Financial toxicity burdens remain understudied in this patient group. The impact of proton therapy on financial toxicity from both the patient-centered perspective and the system-level perspective in head and neck cancer patients requires more accurate measurement, especially relative to photon-based strategies. This will require detailed evidence on the impact of treatment on restoring patients' ability to be productive, cope with lingering disability, and function at work, in households, and in society. Such evidence will be critical for assessing the value of proton therapy and selecting patients who are most likely to benefit from proton therapy for head and neck cancer; thereby maximizing clinical benefit while minimizing financial toxicity in head and neck cancer patients.
